# Targeting drug‐tolerant cells: A promising strategy for overcoming acquired drug resistance in cancer cells

**DOI:** 10.1002/mco2.342

**Published:** 2023-08-24

**Authors:** Xiaohai Song, Yang Lan, Xiuli Zheng, Qianyu Zhu, Xuliang Liao, Kai Liu, Weihan Zhang, QiangBo Peng, Yunfeng Zhu, Linyong Zhao, Xiaolong Chen, Yang Shu, Kun Yang, Jiankun Hu

**Affiliations:** ^1^ Department of General Surgery Gastric Cancer Center Laboratory of Gastric Cancer State Key Laboratory of Biotherapy West China Hospital Sichuan University Chengdu China; ^2^ Department of Radiology Huaxi MR Research Center (HMRRC) and Critical Care Medicine Precision Medicine Center, Frontiers Science Center for Disease‐Related Molecular Network, West China Hospital Sichuan University Chengdu China

**Keywords:** acquired drug resistance, drug‐tolerant cells, phenotype plasticity, translational remodeling

## Abstract

Drug resistance remains the greatest challenge in improving outcomes for cancer patients who receive chemotherapy and targeted therapy. Surmounting evidence suggests that a subpopulation of cancer cells could escape intense selective drug treatment by entering a drug‐tolerant state without genetic variations. These drug‐tolerant cells (DTCs) are characterized with a slow proliferation rate and a reversible phenotype. They reside in the tumor region and may serve as a reservoir for resistant phenotypes. The survival of DTCs is regulated by epigenetic modifications, transcriptional regulation, mRNA translation remodeling, metabolic changes, antiapoptosis, interactions with the tumor microenvironment, and activation of signaling pathways. Thus, targeting the regulators of DTCs opens a new avenue for the treatment of therapy‐resistant tumors. In this review, we first provide an overview of common characteristics of DTCs and the regulating networks in DTCs development. We also discuss the potential therapeutic opportunities to target DTCs. Last, we discuss the current challenges and prospects of the DTC‐targeting approach to overcome acquired drug resistance. Reviewing the latest developments in DTC research could be essential in discovering of methods to eliminate DTCs, which may represent a novel therapeutic strategy for preventing drug resistance in the future.

## Graphical abstract

Drug‐tolerant cells (DTC) have features including slow‐cycling, stem cell‐like, EMT‐like, senescence‐like and diapause‐like changes, and could be the reservoir of multiple resistant mutations. In addition, multiple regulatory mechanisms are related to the maintenance of the DT state, like epigenomic, transcriptional and translational regulation; metabolic changes; signaling pathway activation, antiapoptosis, and tumor microenvironment.

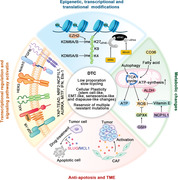

## 1 INTRODUCTION

Cancer remains one of the most serious threats to human health, and its morbidity and mortality rates continuously increase at an alarming rate worldwide. In 2018, 18.1 million newly diagnosed cancer cases and 9.6 million cancer death cases were reported.^1^ Current predominant strategies for cancer treatment include surgery, radiotherapy, and chemotherapy, but these traditional approaches have clinical limitations.^2^, ^3^, ^4^, ^5^ For example, surgery is often not suitable for patients with distant metastases, radiation can induce significant damage to surrounding tissues, resulting in poor wound healing,^6^ and chemotherapy kills both tumor cells and highly proliferative normal cells, leading to systemic toxicity and adverse side effects.^7^ Unveiling molecular mechanisms underlying cancer pathogenesis has prompted effective targeted therapy. Antibodies for key signaling pathways (e.g., epidermal growth factor receptor (EGFR), tyrosine‐kinase inhibitors (TKI), human epidermal growth factor receptor‐2 (HER2)),^8^, ^9^, ^10^, ^11^ inhibitors for vascular endothelial growth factors (VEGF) (e.g., Bevacizumab and Ramucirumab)^12^, ^13^, ^14^ and immune checkpoint inhibitors (e.g., programmed death 1 (PD‐1), programmed death‐ligand 1 (PD‐L1), and cytotoxic T lymphocyte‐associated antigen‐4 (CTLA‐4)),^15^, ^16^ have been explored for cancer treatment,^17^, ^18^, ^19^, ^20^ some of which have revolutionized anticancer therapeutic methods. Unfortunately, despite advances in patient prognosis using the above‐mentioned treatment methods, drug resistance remains a major barrier to cancer treatment.^21^, ^22^, ^23^, ^24^

**FIGURE 1:**
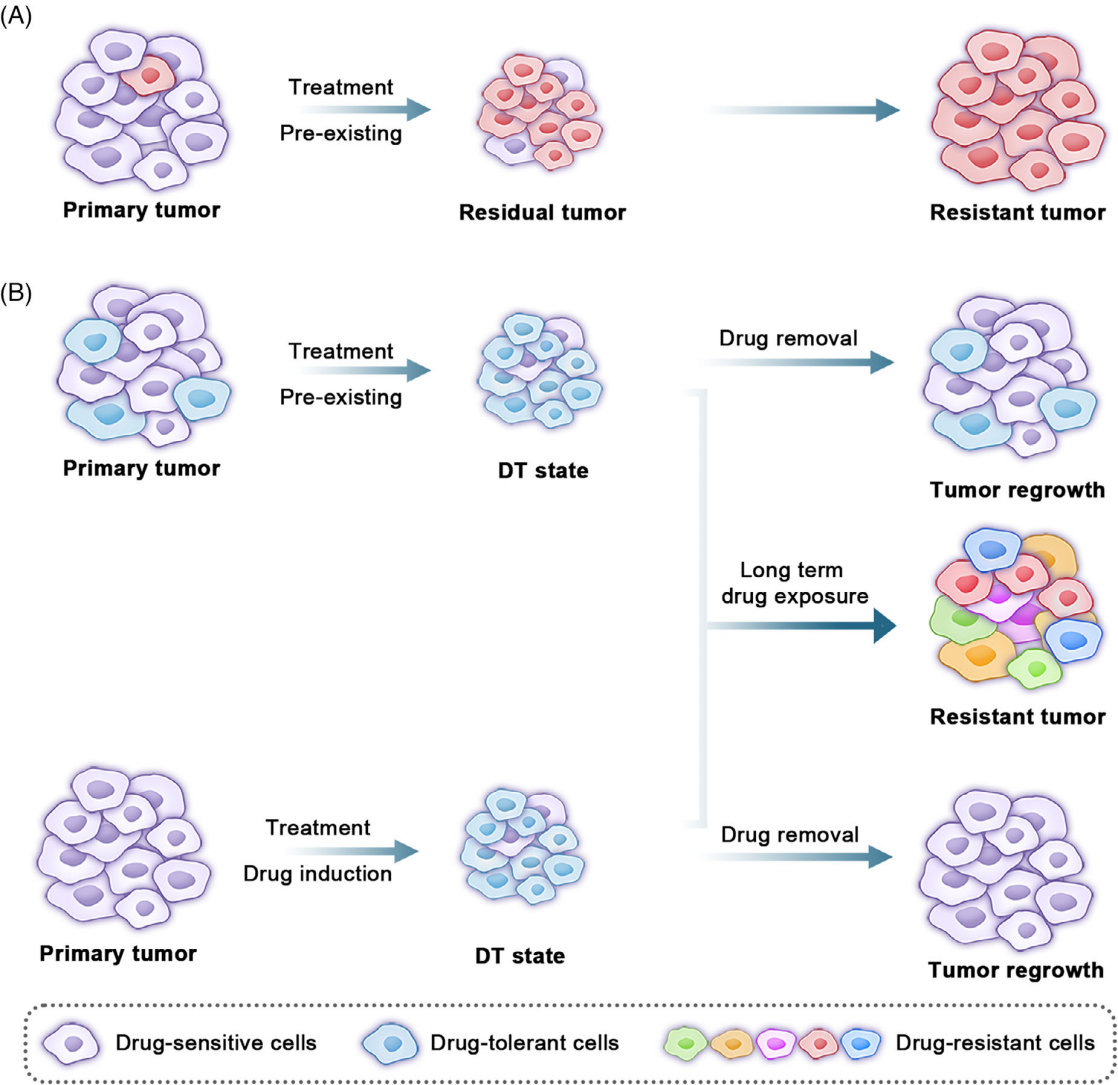
Cancer cells can acquire drug resistance via genetic and nongenetic adaptation mechanisms. (A) A small number of subclones have preexisting or develop new genetic alterations that confer drug resistance, and these drug‐resistant tumor cells survive during the treatment, leading to drug resistance. (B) There are two hypotheses for the source of DTCs and acquisition of tumor resistance. The first (top) is that a subpopulation of cancer cells with DTC characteristics presents in treatment‐naive tumors and it selectively survive from drug exposure (clonal selection); and the second (bottom) is that drug treatment induces a phenotypic transition of tumor cells into the DTCs. DTCs exhibit a reversible phenotype; they can resume proliferation and resensitize their drug sensitivity after termination of drug treatment, and they eventually acquire drug resistance through multiple mechanisms under continuous treatment. DTC, drug‐tolerant cell.

Resistance to cancer therapies is classified as either primary or acquired resistance.^22^, ^25^, ^26^ Primary resistance is that the tumor is initially resistant to drug therapy, which may be due to preexisting genetic alterations that facilitate cancer cells to escape the therapeutic action. In contrast, acquired resistance is that the tumor that responds to the initial treatment becomes insensitive to the same drug after long‐term treatment. Many previous studies have reported the mechanisms of acquired drug resistance in tumors, especially drug resistance in targeted therapy, including genetic drivers of drug resistance such as secondary mutations, activation of bypass signaling pathways, and lineage transformation.^27^, ^28^ In the first resistance mechanism, tumor cells with existing or new genetic alterations that lead to increasing catalytic activity or impairing drug binding to the target oncogene eventually become resistant to targeted therapy (Figure 1A). For example, during the course of gefitinib treatment for EGFR‐mutant lung cancers, a secondary mutation could lead to the conversion of threonine to methionine at the amino acid position 790 in the exon 20 of the EGFR gene, resulting in an increase in the spatial structure of side chains of the target site to enhance steric hindrance to the therapeutic drug. Obstruction of the binding of EGFR TKI to EGFR leads to the reactivation of EGFR with increased affinity to adenosine triphosphate (ATP), which eventually results in the emergence of drug resistance. Secondary mutations have become a leading cause of resistance to first‐ and second‐generation EGFR inhibitors.^29^, ^30^ Similarly, BRAF amplifications or BRAF splicing variants promote resistance to BRAF‐targeted therapy in melanoma.^31^, ^32^, ^33^ In the second resistance mechanism, adaptive signaling pathways are reactivated via mechanisms that are different from the original drug targets, and “bypasses” of oncogenic signaling is conferred to tumor cells, resulting in drug resistance. For example, MET activation, another cause of EGFR TKIs resistance in lung cancer, can promote drug resistance by reactivating PI3K/AKT and MEK/ERK signaling pathways in the presence of EGFR inhibition.^34^, ^35^, ^36^ In the third resistance mechanism, tumor cells are characterized by the transition from one cell identity to another under the pressure of selective therapy. For example, histologic transformation of a lung adenocarcinoma subtype to a small cell carcinoma subtype promotes resistance to EGFR TKIs.^37^, ^38^ Although the molecular mechanism of this lineage transformation remains unclear, it has been shown that certain genomic features are required for this phenotypic transformation.^39^, ^40^

Although many studies have shed light on specific mechanisms of acquired resistance, it remains unknown how resistant clones emerge and evolve during treatment. Traditionally, acquired drug resistance has been attributed to preexistence or de novo acquisition of specific gene mutations (Figure 1B). First, due to clonal heterogeneity within the tumor, a small number of subclones may display existing or new genetic variations (e.g., gene mutation, gene amplification, gene deletion, or chromosomal translocation) to induce drug resistance even before the initiation of therapy. In addition, therapeutic resistance may be developed in the absence of genetic variations.^41^, ^42^, ^43^ It has been reported that a small population of tumor cells can enter a drug‐tolerant (DT) state to escape the intense pressure exerted by therapeutic agents during initial drug treatment and they acquire secondary resistance mutations, resulting in disease progression.^44^, ^45^ Cells in such a DT state are known as drug‐tolerant cells (DTCs) or drug‐tolerant persisters (DTPs). They are initially present in a quiescent state with reduced sensitivity to therapeutic drugs, but resume proliferation and become sensitive to these drugs again after drug removal. The DT state is not associated with cancer cell genetic variants, instead, it is linked to nongenetic variations with a non‐ or slow proliferation status. Reversible properties of DTCs are similar to the phenomenon of “retreatment response” in clinical tumor treatment. However, tumor cells in a DT state can produce stable drug‐resistant clones through a variety of mechanisms during the course of continuous treatment, therefore, they are known as a reservoir of drug‐resistant cells.^46^, ^47^ In the clinic, tumor cells entering the DT state are the root cause of the minimum residual disease (MRD), which occurs at the point of reaching the maximal tumor shrinkage prior to eventual tumor progression.^48^, ^49^, ^50^

Advances have been made in the study of resistance‐inducing genetic variations. Revealing nongenetic adaptive mechanisms could help demystify the early development of acquired resistance, eventually overcoming tumor resistance.^51^, ^52^ In this context, profiling the characteristics of DTCs and unveiling the mechanisms governing the regulation of DT states could aid in discovery of effective methods to eliminate DTCs, which may be a novel therapeutic strategy for preventing acquired drug resistance. Previous studies have reported the general characteristics of DTCs and key regulators of the DT state.^44^, ^45^, ^46^, ^53^, ^54^ In addition, there are two theories about the origin of DTCs.^55^ Furthermore, multiple regulatory mechanisms have been reported to be involved in the maintenance of the DT state.^28^ However, no consensus has been reached regarding the characteristics, origin(s) and regulatory mechanisms of DTCs, particularly in vivo, which have hampered translation of discoveries of DTCs into curative precision therapeutic modalities.

In this review article, we detail the aforementioned common characteristics of DTCs, survey the regulatory mechanisms that regulate the DT state, and discuss therapeutic strategies to target DTCs. The challenges and prospects of targeting DTCs for cancer treatment are provided.

## 2 CHARACTERISTICS OF DTCs

We summarized four common characteristics of DTCs including prevalence in tumors, slow‐cycling, phenotype plasticity, reversible drug sensitivity, and reservoirs for resistant phenotypes.

### 2.1 Prevalence of DTCs in tumors

**FIGURE 2:**
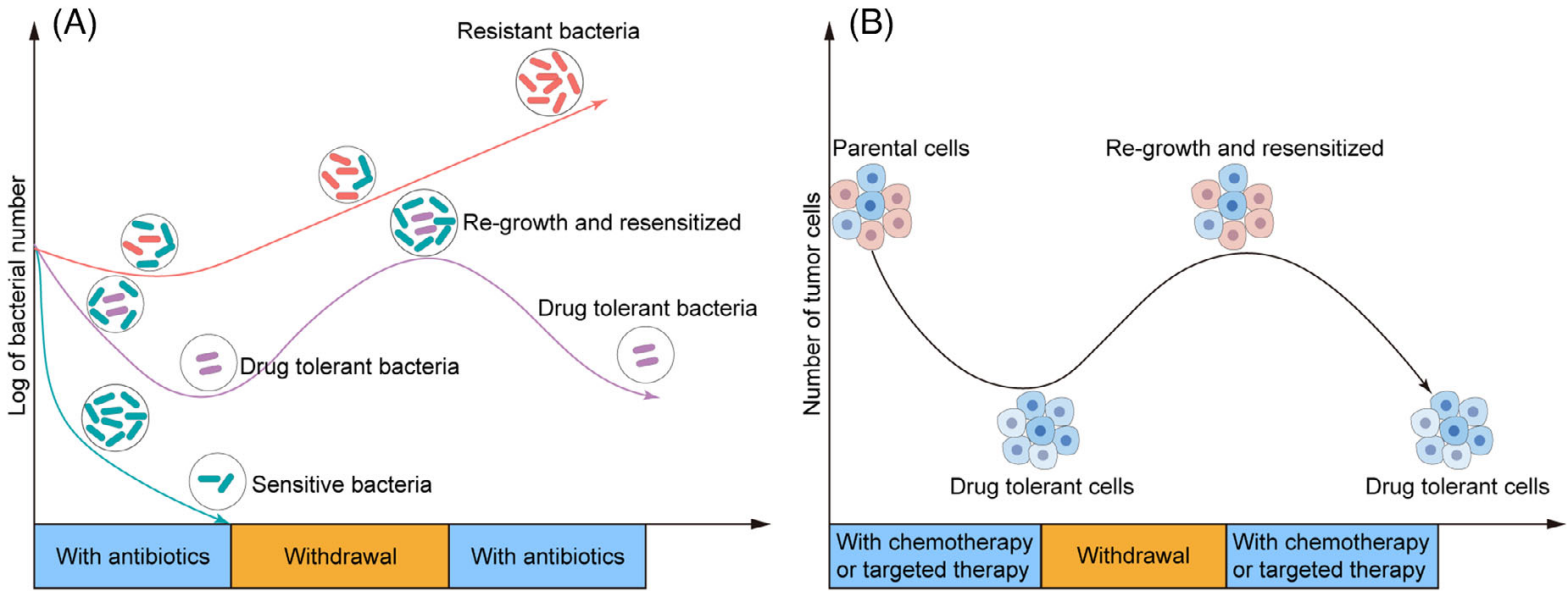
Conceptual similarities of tolerance in bacteria and tumor cell. (A) The survival curve describes the changes in bacterial status when exposed to antibiotics. Sensitive bacteria (cyan) are completely eliminated with antibiotic treatment, while antibiotic‐resistant bacteria (red) continue to proliferate. Antibiotic‐tolerant bacteria can survive when exposed to antibiotics and quickly reproduce after the removal of antibiotics, regaining sensitivity to antibiotics (purple). (B) The survival curve depicts the emergence of drug‐tolerant cells (DTCs) in tumors following chemotherapy or targeted therapy, as well as their reversible characteristics. The hallmark of the tolerant state is its reversibility upon discontinuation of the drug, indicating the absence of resistance‐related mutations.

The concept of DT originated from microbiology and it was referred to the ability of a subpopulation of bacteria to resist antibiotics.^56^, ^57^, ^58^ After the drug is withdrawn, the slow‐growing bacteria in a DT state that have survived antibiotic treatment are able to resume proliferation and reconstruct a drug‐sensitive population, implying phenotypic plasticity, rather than genetic variation, is responsible for mediating transient bacterial drug persistence. Settleman and coworkers^44^ were the first to apply the concept of DT to cancer research in 2010. Their findings revealed that a small subset of PC9 non‐small cell lung cancer (NSCLC) cells survived from erlotinib (an EGFR inhibitor) treatment at a lethal dose. These cells were referred as DTCs that exhibited a low proliferation rate with no identified driver genetic variations (Figure 2). They then examined others cancer cells, including those of melanoma, lung cancer, breast cancer (BC), and colorectal cancer (CRC), and a similar DTC subpopulation was obtained after various drug treatments (cisplatin and EGFR/RAF/MET inhibitors). Since then, the role of DTCs in tumor drug resistance has attracted significant interest and DTCs have been detected in a variety of cancer cell lines, including gastric cancer,^59^ ovarian cancer,^60^ glioblastoma cell lines,^61^ osteosarcoma,^62^ and leukemia^63^ treated with chemotherapy or targeted therapy.

In addition to their presence in tumor cell lines in vitro, DTCs have been recently detected in vivo in cell‐ and patient‐derived xenografts (CDX and PDX, respectively). DCTs have been reported to be responsible for disease relapse after drug withdrawal in a CDX model of basal cell carcinoma (BCC).^64^, ^65^ In a PDX model of triple‐negative breast cancer (TNBC), Echeverria et al.^53^ found that a subpopulation of tumor cells in a DT state was able to repopulate tumor and such a subpopulation was also reported by Vallot and coworkers.^66^ In addition, in a PDX model of CRC, O'Brien and coworkers^67^ discovered that the response of the PDX to FOLFIRI and CPT‐11 was the same as that of a tumor cell line in a DT state in vitro. These studies support that the emergence of DT is independent of a cancer type or the modality of therapeutic treatment. Tumor cells could enter the DT state after initial drug treatment to escape drug killing, both in vivo or in vitro, which provides great insights into developing strategies to overcome acquired resistance through targeting DTCs.

### 2.2 Proliferation‐ slow‐cycling, quiescence, and dormancy

The most distinct feature of DTCs is their slow proliferation rate or the absence of proliferation, known as dormancy or quiescence.^44^, ^68^ Cellular dormancy often refers to a reversible nonproliferating state of a cell which has undergone the G0–G1 cell cycle arrest. Dormancy and quiescence share great similarity and cell dormancy is usually achieved by activating a quiescent program. Therefore, dormant cells are often referred as quiescent cells. Entering a dormant state may contribute to evolution in survival mechanisms, so that tumor cells could survive after long‐time drug exposure.^28^, ^69^

The slow‐cycling or dormant cancer cells are found to be similar to cancer stem cells (CSC).^69^ DTCs reported in a few articles have a stem‐like phenotype, which is characterized with a quiescent slow‐proliferation state, helping them survive from drug treatment or new environments.^44^, ^55^, ^61^, ^70^, ^71^, ^72^, ^73^ Internal and external factors, such as drug treatment, can induce cellular stress, resulting in senescence and a shift to a slow‐cycling state. Typically, the senescence‐like phenotype induced by stress response is identified as a biological characteristic of DTCs.^74^, ^75^, ^76^ In addition, it has been demonstrated that DTCs exhibit biological and transcriptional characteristics of diapause, which is defined as a stage of dormancy for embryonic cells induced by stress and characterized by a slow‐cycling phenotype.^67^, ^77^

Although it has been found that there are different phenotypes of DTCs, they share a common characteristic of slow cycling. Slow cycling can help tumor cells evade tumor treatments that prevent cell proliferation and induce apoptosis. Moreover, drug resistance can be developed through dormancy, resulting in the mutation and remodeling of epithelial cells in the tumor into a proliferative state, and eventually rapid tumor growth and recurrence. The mechanism for slow proliferation of DTCs is not fully unveiled. It has been reported that slow proliferation may be regulated by epigenetic reprogramming, transcriptional regulation, or interaction with the tumor microenvironment (TME).^78^

### 2.3 Phenotype plasticity

Cancer cell plasticity refers to the ability of cancer cells to switch between different phenotypes in response to external stimuli. This phenotypic switch of cancer cells has been identified as an escape mechanism for selective drug treatment and this switch disappears after a drug‐free interval.^79^ It has been reported that DTCs undergo transient CSC‐like, epithelial‐to‐mesenchymal‐like, senescence‐like, and diapause‐like changes in response to drug stress.^80^, ^81^, ^82^

CSCs are a small group of tumor cells with an infinite proliferation potential and a self‐renewal ability. They can differentiate into heterogeneous tumor cells, which is considered to be one of the root causes of tumor development, recurrence and metastasis, and drug resistance.^63^, ^83^ Previous studies reveal that CSCs and adult stem cells share multiple signatures, including the surface markers of CD133, CD44, CD24, CD26, CD166, and EPCAM, and intracellular proteins like ALDH1.^84^, ^85^, ^86^, ^87^, ^88^ Additionally, CSCs and stem cells also share similar development‐related signaling pathways, such as WNT, NOTCH and Hedgehog (Hh).^89^, ^90^ CSCs are characterized by slow proliferation or a quiescent state, thus they become resistant to antiproliferation drugs. Cancer cells have been reported to switch to a stem cell‐like phenotype in a variety of tumors after chemotherapy. Several stem cell markers, such as CD24, CD44, CD133, ALDH1, CD271, Oct‐4A, Sox2, and ATP‐binding cassette efflux transporters, are found to be highly enriched in DTCs from lung cancer, melanoma, BC, GC, and bladder cancer.^44^, ^59^, ^71^, ^91^, ^92^, ^93^ These findings suggest that CSC‐like phenotype acquisition facilitate the development of a highly plastic tolerant state in DTCs. However, several studies have indicated that the enrichment of leukemia stem cells are not found from acute myeloid leukemia (AML) cells that survive from chemotherapy.^94^, ^95^, ^96^ Similarly, TNBC tumors do not display an enrichment of stem‐like cells after neoadjuvant chemotherapy,^53^ which suggest that the stem cell‐like phenotype is not a common characteristic of DTCs but it is induced under drug stress conditions.

Epithelial–mesenchymal transition (EMT) refers to the process that cells lose epithelial characteristics and gain mesenchymal features,^97^ while mesenchymal–epithelial transition (MET) is the inverse process of EMT. It has been reported that EMT and MET are regulated predominantly at the transcriptional level by the activity of EMT‐transcription factors (EMT‐TFs), including Snail, Slug, Zeb1/2, Twist, and microRNAs (miRNAs).^98^, ^99^, ^100^ Signaling pathways such as transforming growth factor β (TGF‐β), WNT, NOTCH, and HIPPO are also involved in this process.^101^, ^102^, ^103^, ^104^ EMT is a powerful source of functional and phenotypic plasticity, and cancer cells can utilize this EMT process to enhance their adaptability to a DT condition.^47^, ^105^, ^106^ It endows cells with the ability to metastasize and invade, reduce apoptosis and senescence, and promote immunosuppression, which not only plays a key role in development but is also involved in processes such as tissue healing, organ fibrosis, and cancer.^107^, ^108^, ^109^ In addition, previous studies have confirmed that EMT programming contributes significantly to the development of resistance to various types of therapeutic agents in multiple cancer types.^110^, ^111^, ^112^, ^113^ Inhibition of EMT can effectively eliminate cisplatin resistance in ovarian cancer.^114^ Transcriptomic analysis of EGFR‐mutant lung adenocarcinoma cells^47^ and patient‐derived melanoma models treated with targeted drugs^115^ reveals that the EMT signature is enriched in DTCs. In addition, EMT‐related biomarkers, such as vimentin, Zeb, Twist, and Slug, are upregulated in DTCs derived from EGFR‐mutant lung cancer cells after EGFR TKI therapy.^74^, ^116^, ^117^ Furthermore, activation of the EMT program is also been found in other DTC models subjected to diverse treatment regimens.^60^, ^76^, ^118^ Accordingly, these results suggest that DTCs may experience adaptive plasticity as a result of the EMT process.^81^

A proportion of surviving DTCs exhibit senescence signs after drug withdrawal, such as H3K9Me3 positive nuclear foci, activation of the CDK inhibitor 1B (p27Kip1), and an increase in the senescence‐related ß‐galactosidase activity.^74^ Kunimasa et al.^91^ reported that senescence‐related proteins in DTCs from PC9 cells treated with EGFR TKI, such as γH2AX, pRb, p21, and p27, are upregulated. In line with the above findings, one recent article discovered that SASP/inflammatory transcriptomic and senescence hallmarks are enriched in DTCs in preclinical models of CRC and AML.^67^ Similarly, cells collected from melanoma and AML cases exhibits a senescence‐like phenotype after TKI treatment and chemotherapy.^94^ Thus, senescence can be considered as the conserved mechanism of cellular responses to treatment stress. In addition to the senescence‐like phenotype, DTCs have been reported to exhibit transcriptional and biological features of diapause, a stress‐induced dormant state of embryonic cells to cause delayed development.^94^ DTCs isolated from CRC xenografts and BC‐derived organoids have been found to undergo an unsaturated invertible diapause‐like program, and they display a reduced Myc activity and a slow‐cycling phenotype.^67^, ^77^

DTC shows a variety of phenotypic changes, and the specific markers of these phenotypes will help us to find DTC in tumor tissues. Targeting the potential vulnerability of DTCs related to their phenotype plasticity may open a new door for enhancing the efficacy of drugs against residual diseases at the clinical level.

### 2.4 Reservoirs of resistant cell clones

In contrast to drug resistance, a state of tolerance refers to temporary “tolerance” to a drug: the drug does not induce cell death, but exerts an inhibitory effect. When the drug is withdrawn for a period of time, the sensitivity to the tumor cells can be restored. However, several studies have shown that DTCs can acquire stable resistance through multiple mechanisms during long‐term drug exposure. Hata et al.^47^ used gefitinib, an EGFR inhibitor, to treat PC9 cells until resistant clones were developed. During the process, they majority of cells initially die, while the remaining small subsets of surviving DTCs expand slowly, eventually they develop complete drug resistance after 1 year of treatment. Subsequent results reveal that DTCs without the original EGFRT790M cells would eventually have the EGFR T790M mutation or develop other genomic resistance mechanisms, demonstrating de novo evolution of the DTC resistance mechanisms. In a PDX model of CRC, after CPT‐11 is added to treat mice for 5−6 months; genetically resistant clones are developed from tumors in a DT state.^67^ Similar findings are obtained by Altschuler and coworkers,^46^ who derived a panel of erlotinib‐resistant cell lines from one single DTC of PC9 cells. Collectively, these resistant cells recapitulate multiple mechanisms of acquired resistance observed in the clinic, including EGFR T790M mutation and MET amplification. These results suggest that targeting DTCs before they develop resistance mutations has the potential to mitigate or overcome drug resistance.

**FIGURE 3:**
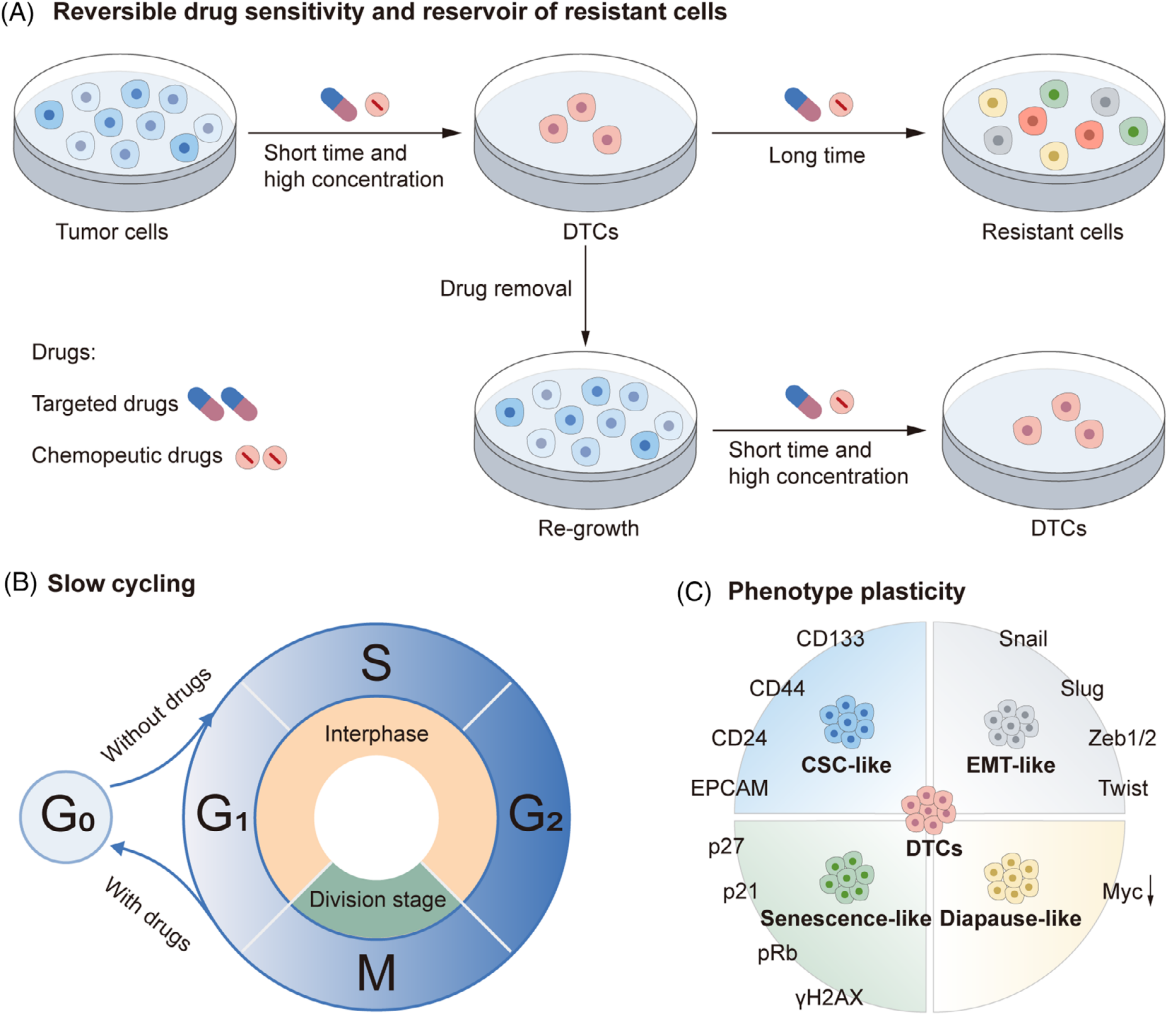
The biological characteristics of DTCs. The features of DTC including reversible drug sensitivity and reservoir of multiple resistant mutations (A), slow cycling (B), and phenotype plasticity (CSC‐like, EMT‐like, senescence‐like and diapause‐like changes) (C). CSC, cancer stem cells; EMT, epithelial–mesenchymal transition; DTC, drug‐tolerant cell.

**FIGURE 4:**
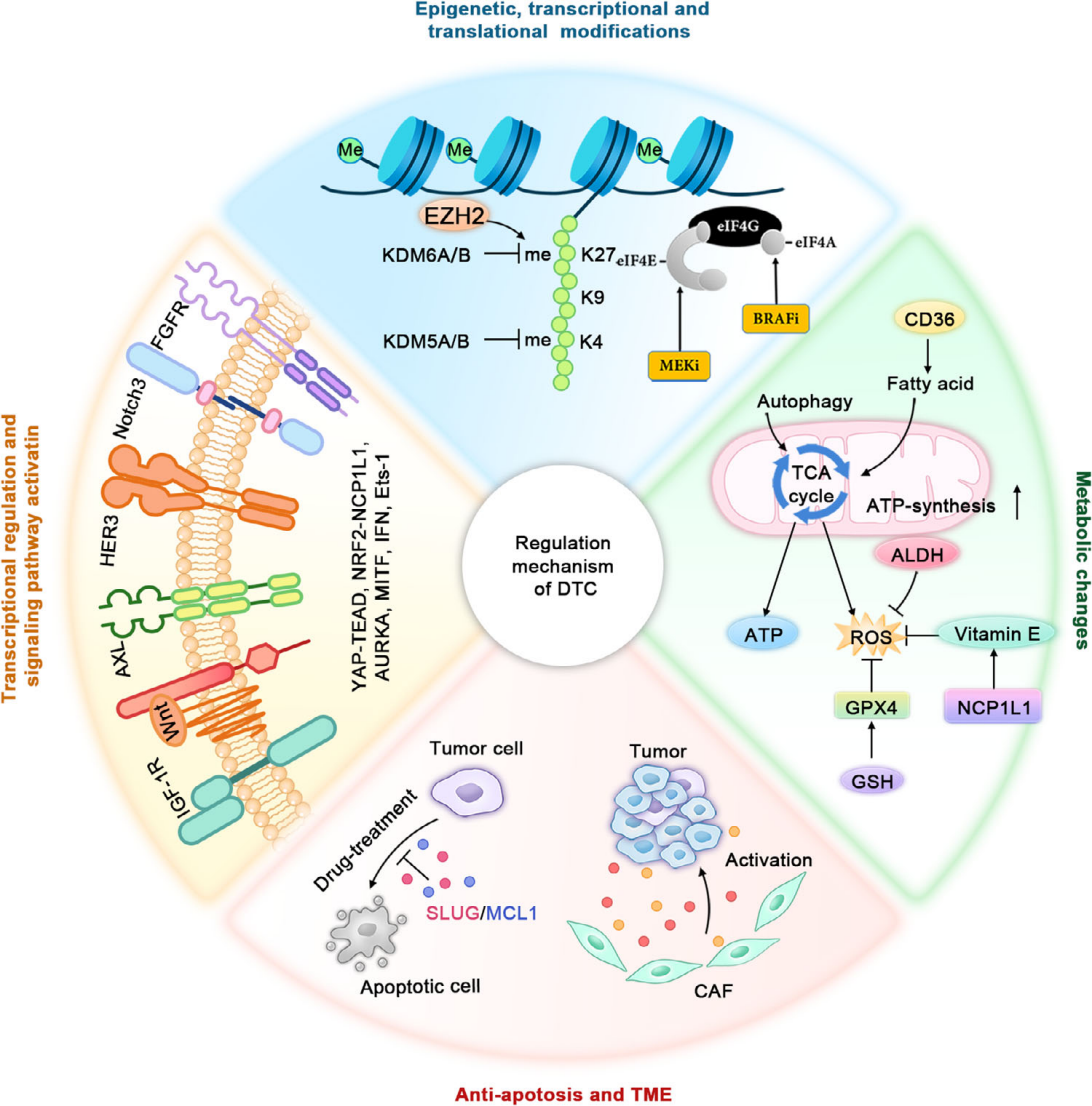
The molecular mechanisms associated with DTCs. The regulation mechanisms of the DT state: (1) Epigenomic, transcriptional and translational regulation: DTCs usually display upregulation of demethylases such as KDM5 and KDM6, which reduces histone H3K4me and H3K27me. In addition, transcriptional and translational regulation is observed in DTCs; (2) metabolic changes: DTCs are dependent on mitochondrial respiration for their energy production. Fatty acid β‐oxidation and autophagy contribute to this energy production. DTCs have an antioxidant stress capacity via upregulation of GPX4, ALDH, and NCP1L1 to protect them against the oxidative stress‐induced cytotoxicity; (3) signaling pathway: activating alternative and bypass pathways, such as IGF‐1R, AXL,FGFR3, and HER3 pathways, play an important role in DTC survival; and (4) antiapoptosis and tumor TME: DTCs have the ability to resist drug‐induced apoptosis via upregulation of SLUG and MCL‐1 and drug treatment affects microenvironmental nontumor cells such as CAFs to promote DTC formation. CAF, cancer‐associated fibroblast; DTC, drug‐tolerant cell; EMT, epithelial–mesenchymal transition; FGFR, fibroblast growth factor receptor; HER3, human epidermal growth factor receptor 3; IGF‐1R, insulin‐like growth factor 1 receptor; TME, tumor microenvironment.

Currently, there is no consensus on how to define the DT cell state. The common characteristics of DTCs may be the foundation for the definition of DTCs. Four characteristics are essential for defining DTCs including slow‐cycling of the surviving cells upon treatment, decreased sensitivity of the DTCs toward anticancer agents, reversible cell proliferation and regained drug sensitivity to the same treatment, and genetic resistance upon continuous anticancer treatment^78^ (Figure 3). These characteristics could be used to differentiate DTCs from other cell states, such as cancer cell dormancy, caner stem cells, senescence, and diapause phenotypes. In fact, these biological features like slow cycling and cell plasticity are not mutually exclusive; on the contrary, they can coexist in DTCs and they are regulated via multiple mechanisms, such as epigenetic modifications, transcriptional regulation, mRNA translation remodeling, metabolic changes, activation of signaling pathways, antiapoptosis, and modulation of the TME (Figure 4).

## 3 KEY MOLECULAR PROCESSES IN DTCS

### 3.1 Epigenetic modifications and transcriptional regulation

Epigenomic reprogramming refers to heritable changes in gene expression without changes in the DNA sequence, which mainly involves DNA methylation, histone modification, noncoding RNA (ncRNA) alteration and chromatin remodeling.^119^ In general, DNA methylation often affects gene expression, transcription, and protein activity. Hypermethylation of the cytosine‐guanine sequence (CpG) islands usually leads to transcriptional inhibition and a decrease in the gene expression.^120^ In addition, histone acetylation is closely related to gene transcription and an alteration in histone acetylation may lead to the development of cancer phenotypes.^121^, ^122^, ^123^, ^124^ Epigenetic changes, particularly abnormal modifications of histones or genomic DNA, can lead to chemotherapeutic resistance.^125^, ^126^ Recently, epigenetic changes have been considered to be crucial in acquiring the DT phenotype. It has been well accepted that the globally repressed chromatin state caused by histone methylation, demethylation or acetylation may contribute to the formation of DTCs.

H3K4 trimethylation, a biomarker of active transcription, plays a critical role in stem cell differentiation during development.^127^, ^128^ Demethylases (KDM5A/JARID1A and KDM5B/JARID1B) can specifically remove trimethylation markers on the histone H3K4 to prevent trimethylation. KDM5A and KDM5B have been found to be highly expressed in many cancer cells and these overexpressed enzymes can promote drug resistance.^129^ For example, in temozolomide‐resistant glioblastoma cells, KDM5A expression is significantly boosted, while the inhibition of KDM5A expression promotes apoptosis.^68^ In breast tumors, KDM5A induces PI3K/AKT/mTOR activation by activating IGF1R and Her2 signaling pathways, leading to tamoxifen resistance.^130^ Similar to KDM5A, KDM5B is found to be highly expressed in cisplatin‐resistant gastric cancer cells, which could recruit X‐ray Repair Cross Complementing 1 (XRCC1) through demethylation of H3K4, resulting in cisplatin resistance.^131^ In addition to gastric cancer, KDM5B is also highly expressed in other drug‐resistant tumor cells (such as endometrial cancer cells, melanoma, and NSCLC) and it promotes drug resistance.^132^, ^133^, ^134^, ^135^ DTCs often exhibit a deficiency of H3K4me3 or upregulation of KDM5A^44^, ^136^, ^137^ and/or KDM5B.^70^, ^71^, ^135^, ^138^, ^139^ Furthermore, genetic knockdown of the genes for two demethylases is reported to inhibit DTC generation and reverse the sensitivity of tumor cells to targeted therapy in vitro and in vivo.^70^ In addition, KDM5 inhibitors, such as ryuvidine and CPI‐455, can enhance global H3K4 trimethylation and reduce DTCs in different tumors, such as melanoma, NSCLC, colon cancer and BC, thus restoring the sensitivity of cancer cells to chemotherapy or targeted therapy.^140^, ^141^ These studies reveal that H3K4 demethylation promotes DTC formation, whereas H3K4 methylation impairs DTC formation.

Downregulation of H3K27me3 is also detected in DTCs from melanoma, colon cancer, BC, lung cancer, and glioblastoma.^61^, ^71^, ^142^ KDM6A (UTX) and JMJD3 (KDM6B) discovered in 2007 can demethylate H3K27 and they play an important role in drug resistance.^143^ For example, in chronic myelogenous leukemia, KDM6A could enhance the expression of TRKA through YY1, leading to cancer cell resistance to imatinib.^144^ In addition, PCGF1 can reduce the H3K27me3 level by upregulating the expression of KDM6A, thus activating the transcription of stem cell markers and promoting stem cell proliferation.^145^ Similarly, KDM6B is highly expressed in various cancers and overexpressed KDM6B could promote drug resistance.^146^, ^147^ In neuroblastoma, KDM6B can activate the CDK4/6‐pRB‐E2F pathway via H3K27me3‐dependent enhancer–promoter interaction, thereby promoting palbociclib resistance.^148^ In diffuse large B‐cell lymphoma, inhibition of KDM6B enhances the sensitivity of tumor cells to chemotherapeutic drugs.^149^ Besides, in BC, inhibition of KDM6B induces apoptosis and significantly boosts the sensitivity of cancer cells to GDC‐0941.^150^ Interestingly, upregulation of KDM6A and KDM6B is also observed in DTCs from glioblastoma.^61^ Marsolier et al.^66^ reported that the expression procedure in a DT state in untreated TNBCs could be ascribed to H3K4me3 and H3K27me3. Furthermore, H3K27me3 depletion enhances tumor cell chemoresistance. On the contrary, suppression of H3K27me3 demethylation during chemotherapy prevents the conversion from an active proliferation state to a DT state.^66^ H3K27me3 expression, on the other hand, help formation of DTCs in targeted therapy‐treated lung cancer.^45^ EZH2 inhibition, which results in the loss of H3K27me3, helps reduce the number of DTCs, indicating that the effect of H3K27 demethylation on drug tolerance may be associated with the type of the tumor.^151^ These data suggest that inhibition of KDM6A and/or KDM6B could become an effective therapeutic strategy to overcome drug resistance.

The melanoma and glioblastoma‐derived DTC models have shown an increased level of H3K27 acetylation,^61^, ^152^ while DTCs derived from lung cancer display globally reduced H3K acetylation.^44^, ^45^ Trichostatin A, an inhibitor for histone deacetylase (HDAC), in combination with targeted therapy, has been reported to contribute to the selective ablation of DTCs derived from lung cancer in vivo and in vitro^44^, ^45^; however, Trichostatin A could not eliminate melanoma‐derived DTCs after exposure to targeted therapy.^153^

H3K9me3 accumulation has been identified as a frequent biomarker of DTCs.^71^, ^74^, ^92^, ^142^, ^154^, ^155^ H3K9me3 has previously been associated with the silencing of tumor suppressor genes, thus H3K9me3 upregulation could be a strong predictor of poor prognosis in a variety of cancers.^156^, ^157^, ^158^ H3K9me3 hypermethylation is due to an increased level of methyltransferases, such as KDM1B, SETDB1, and SETDB2. Knockdown of these methyltransferases significantly reduces the level of H3K9me3, while boosts the treatment sensitivity of DTCs.^45^, ^71^, ^142^ In addition, DTC occurrence has been reported to be influenced by HP1g, H3.3 and ATRX, which promote the H3K9me3‐induced generation of heterochromatin. Similarly, DTCs from exhibit a repressed chromatin state characterized by increased H3K9 and H3K27 and its survival is partly maintained by regulators of H3K9me3‐mediated heterochromatin formation.^45^

Structural alterations in the chromatin may induce remodeling of enhancers or super‐enhancers, which regulates the transcriptional programs, thereby affecting transcription.^159^, ^160^ Epigenetic readers, such as bromodomain and extraterminal (BET) proteins, have been reported to promote transcriptional adaptation and chromatin remodeling that are essential for establishing a DT state under some circumstances. For example, BET proteins facilitate chromatin responses to PI3K and MEK inhibitors in BC models, whereas JQ1, a BET inhibitor, has an opposite effect.^118^, ^161^ Other master regulatory factors for critical developmental processes, such as the enhancer of zeste homologue 2 (EZH2), a catalytic subunit of Polycomb repressive complex 2, have emerged as an important driver of epigenetic alterations that lead to drug resistance. Comparison of proteome‐wide lysine methylation profiles reveals that the methylation level of DTPs increases on K116 of the PRC member Jarid2 in BC and lung cancer, and it is similar to that of EGFR inhibitor‐treated EGFR‐mutant NSCLC cells, thereby facilitating PRC2 stabilization and recruitment into chromatin.^162^ Apart from methylation of the histone H3K27, EZH2 is also responsible for methylating G9a at K185, thereby enhancing recruitment of the repressive G9a complex into chromatin. EZH2 exerts an important effect on different epigenetic alterations indirectly by regulating additional chromatin remodeling enzymes or directly by histone methylation, thereby enabling DTP generation. Targeted therapeutic treatments with CDK4/6 and EGFR inhibitors have been reported to potently reactivate repetitive elements within tumor cells, leading to cell death.^45^, ^163^ In a previous study, RNA‐sequencing suggests that after TKI exposure, the levels of interferon (IFN)/antiviral response genes and LINE‐1 elements are upregulated in most EGFR‐mutant NSCLC cells.^45^ Nonetheless, H3K9 methylation accumulates in the LINE‐1 elements of DTCs, preventing gene upregulation after drug exposure and improving cell survival. HDAC inhibitors can prevent heterochromatin formation mediated by H3K9 methylation, thereby de‐repressing LINE‐1 elements in DTCs and reinducing cell death. Consequently, inhibition of repetitive elements through a suppressive epigenetic remodeling‐enhanced chromatin environment may provide a new mechanism for persister cells to evade drug treatment effects.

As one of the important components of epigenetics, ncRNAs have been demonstrated to be important regulatory factors for every cellular process. They can affect gene expression and act as oncogenes or tumor suppressors in a variety of tumors.^164^, ^165^, ^166^ Increasing evidence suggests that miRNAs play an important role in epigenetic regulation of various phenotypic states of cancer cells.^167^, ^168^, ^169^, ^170^ miRNAs could influence genetic programs through posttranscriptional silencing of target genes either by promoting translational repression or degrading the target mRNA.^171^ miRNAs have been implicated in regulation of various aspects of cancer biology, including drug resistance, cancer cell stemness, EMT and metastasis.^172^, ^173^ In addition, miRNAs have been reported to be involved in the regulation of DTC formation. For example, peroxyedoxin 6P (RDX6) can promote the formation of DTCs by regulating the PLA2/PKCa activity and reactive oxygen species (ROS), while miR‐371‐3p can specifically bind to PRDX6 to reduce its expression level and inhibit the formation of DTCs.^174^ Furthermore, miR‐147b is significantly upregulated in osimertinib‐tolerant lung cancer cells, and it could promote the formation of osimertinib‐tolerant cells through mediating TCA cycle dysfunction and pseudohypoxia.^153^ These reports suggest that miRNA plays a double‐edged role in the formation of DTCs.

DNA methylation represents a crucial process in epigenetic reprogramming and has a critical effect on tumor development and drug resistance. DNA methylation is coregulated by DNA methyltransferases and demethylases, which can modulate drug resistance in tumor cells mainly through regulating apoptosis, stemness, EMT, and cell proliferation through Notch and Wnt/β‐catenin signaling pathways.^175^, ^176^, ^177^, ^178^, ^179^, ^180^, ^181^ For example, methylation of the brain‐expressed Xlinked protein 1 (BEX1) mediated by DNMT1, one of DNA methyltransferases, can maintain the self‐renewal capacity of liver stem cells by activating the Wnt/β‐catenin signaling pathway, leading to drug resistance.^175^ In addition, DNMT1‐induced hypermethylation of the miR‐34a promoter region activates the Notch signaling pathway, leading to a decrease in the sensitivity of pancreatic cancer cells to sorafenib, while treatment with decitabine, a small molecular inhibitor of DNMT1, enhances the sensitivity of pancreatic cancer cells to sorafenib.^176^ Given the important role of DNA methylation in drug resistance, targeting DNA methylation is considered to be a promising therapeutic approach to overcoming drug resistance. A number of small molecular inhibitors targeting DNA methylation have been developed. Among them, azacytidine, guadecitabine (SGI‐110), and decitabine as DNMT inhibitors have entered clinical trials.^182^, ^183^ However, genome‐wide DNA methylation specific to the CpG site does not induce any distinct alteration in the DT state.^142^ These findings suggest that different histone methylations but not DNA methylation could act as the driver for conversion from parental cells into DTCs.

### 3.2 mRNA translation remodeling

Translational remodeling of mRNAs during gene expression can lead to rapid, specific changes in cancer phenotypes, which allow cells to rapidly and dynamically adapt to a variety of stimuli, including aberrant oncogenic signals, drug killing effects, and microenvironmental stresses.^184^ Some tumor cells are able to overcome drug treatment by undergoing translational reprogramming during translation initiation and elongation. Components of the eIF4F complex are well known to mediate resistance to a variety of cancer treatments, including chemotherapeutics and targeted therapy.^185^, ^186^, ^187^, ^188^, ^189^ Recently, in BRAF^V600^ mutant melanoma, a subpopulation of persister cells was discovered to survive through reversible remodeling of mRNA translation after treatment with BRAF and MEK inhibitors.^190^ During this process, a subset of mRNAs was efficiently translated, which leads to persister cell survival in an eIF4A‐dependent manner. Inhibition of EIF4A via silvestrol, an inhibitor of the eIF4A RNA helicase component of the eIF4F translation initiation complex, could abolish efficient translation by the subset of mRNAs and kill melanoma persister cells. These findings suggest that a combination of eIF4A inhibitors and BRAF/MEK inhibitors could effectively suppress cancer development and eradicate persister cells.

### 3.3 Metabolic changes

After drug treatment, DTCs may alter their metabolism for their adaption to new environmental conditions. Tumor cells are usually dependent on anaerobic glycolysis to fuel growth, whereas DTCs are increasingly dependent on mitochondrial respiration to produce energy. In a melanoma with BRAF mutation, slow‐cycling persister cells display an increased level of mitochondrial oxidative‐ATP‐synthetic gene expression after treatment as well as improve their susceptibility to mitochondrial respiratory chain inhibition.^70^, ^191^ Such mitochondrial respiration dependence is detected in DTCs derived from various types of cancer, such as NSCLC, BC, and GC.^53^, ^59^, ^192^ In the DTCs derived from these types of cancer, the increased levels of ROS and hydrogen peroxide are ascribed to fatty acid (FA) oxidation and mitochondrial respiration hypermetabolism.^59^, ^66^, ^70^, ^193^, ^194^, ^195^, ^196^, ^197^, ^198^ The mitochondria are the main site to produce ROS; therefore, changes in mitochondrial oxidative respiration induce oxidative stress in persister cells. DTCs can resist oxidative stress by consuming the excessive amounts of oxygen‐containing molecules via lipid peroxide and superoxide.^199^

Glutathione (GSH) plays an essential role in the inhibition of ferroptosis as well as the detoxification of ROS and xenobiotics after dioxygen and glucose decomposition.^200^, ^201^ Furthermore, GSH contributes to the ROS balance generated during the ATP generation process, resulting in the formation of oxidized GSSG to prevent injuries of oxidative phosphorylation (OXPHOS) to proteins, DNA, and lipids.^202^ DTCs have been found to upregulate the expression of GSH peroxidases, such as GPx2 and GPx4, which are capable of catalyzing oxidative stress detoxification and preventing cell membrane decomposition via lipid peroxidation.^60^, ^197^, ^200^, ^203^ Targeting the GSH metabolism at different levels can affect the GPx4 activity, whereas the inhibition of GPx4 or a deficiency in GSH leads to ferroptosis in DTCs.^60^, ^204^, ^205^, ^206^ Debasish et al. found that ALDH could mitigate the toxicity induced by an increased level of ROS, whereas pharmacologically disrupting the ALDH activity results in ROS accumulation to the toxic level, thus inducing DNA injury and apoptosis in DTCs. Wei et al. found that NPC1L1 could compromise oxidative stress in multidrug‐resistant (MDR) cancer cells by promoting vitamin E uptake during chemotherapeutic/verapamil treatment.^198^

Recent studies have suggested that the success in the treatment of induced senescent cells depends on their metabolic reprogramming related to senescence which is characterized with a hypermetabolic phenotype and an elevated level of glycolysis.^207^ DTCs have been reported to display an increased level of glucose transporters GLUT1/GLUT3 together with an enhanced activity of glycolytic enzyme hexokinase 2, demonstrating a high glycolytic activity in these cells.^91^ Furthermore, inhibiting the glucose metabolism boosts drug sensitivity to tumor cells and prevents DTC formation, implying that the glucose metabolism may be tumor specific.

Remodeling of lipid metabolic pathways also aids in energy generation in the DTCs. Persistent tumor cells have been found to undergo β‐oxidation of FAs, which is an important energy generation pathway of the mitochondrial respiratory chain.^208^, ^209^ After the treatment with a MAPK inhibitor, CD36, an FA transporter, is upregulated and its expression is maintained in the remaining BRA‐mutant melanoma cells.^208^ Similarly, CD36 is overexpressed in AML cells, along with an increased level of b‐oxidation of FAs, activating mitochondrial OXPHOS.^96^

Autophagy is a sensor of the metabolic state, which can be used to evaluate how cells adapt to the environmental demands. For instance, autophagy can promote the generation of metabolic substrates, such as FAs, in the mitochondria by enhancing recycling of metabolic substrates and thus accelerating OXPHOS in tumor cells.^210^ In pancreatic ductal adenocarcinoma, mitochondrial respiration is influenced by autophagy and peroxisomal FA b‐oxidation.^209^ In *Kras* ^G12D^‐driven lung cancer, autophagy is found to promote the generation of metabolic substrates via macromolecular degradation, thus providing nutrients for the mitochondrial TCA metabolism.^211^ Similarly, PINK1‐mediated mitophagy governs DTCs generation from lung adenocarcinoma treated with a MAPK inhibitor via promoting OXPHOS and maintaining redox homeostasis.^212^ A recent study suggests that DTCs that express AXL display a higher autophagic flux after erlotinib treatment.^117^ In CRC models, the embryonic diapause‐like DTCs are significantly impacted by the mTOR‐mediated autophagy program after chemotherapy.^67^ In addition, Sanduja et al.^213^ reported a variety of tumors could acquire tolerance to Ras‐Raf pathway inhibitors through activation of autophagy, which is mediated by the cellular energy sensor AMP‐activated protein kinase.

### 3.4 Signaling pathways

Bulk RNA‐sequencing has been used to detect global transcriptional remodeling in DTCs collected from melanoma, lung cancer and osteosarcoma models.^62^, ^74^, ^214^ In some in vitro models, apoptosis‐ and autophagy‐related gene levels are dysregulated in the DT state.^47^, ^214^, ^215^ After drug treatment, tumor cells are known to regulate the expression level of genes for proteins involved in pathways that promote cell survival. Accumulating evidence suggests that activating alternative and bypass pathways, such as IGF‐1R, AXL, fibroblast growth factor receptor 3 (FGFR3), Notch3, Wnt, and HER3 pathways, could have an important effect on DTC survival.

The IGF‐1R signaling pathway is activated in a variety of tumors and it plays a critical role in tumor progression and chemotherapy resistance.^216^, ^217^, ^218^, ^219^ DTCs collected from PC‐9 after TKI treatment have been reported to maintain the cell viability via the IGF‐1R pathway.^44^, ^220^ Relative to the corresponding parental PC‐9 cells, DTCs highly express the IGF‐1R protein and exhibit a high phosphorylation level. Furthermore, the IGF‐1R pathway can be modulated at various levels (such as ligand, IGF‐binding protein, and receptor). Since IGF‐binding protein 3 (IGFBP3) expression generally increases in DTCs, treatment with an IGF‐1R inhibitor leads to selective ablation of DTCs.^44^ Single‐cell RNA‐sequencing reveals transcriptional differences in metastatic human BC cells: the expression of the IGF1 receptor (IGF1R) increases in DTCs rather than stressed or untreated cells.^136^

Previous studies have confirmed that AXL kinase is heavily involved in tumorigenesis, metastasis, and drug resistance of many cancers.^221^, ^222^, ^223^, ^224^, ^225^ According to a recent study, AXL is stimulated by osimertinib after the termination of a negative feedback loop into SPRY4 in EGFR‐mutant lung cancer cells, resulting in the expression of HER3 and EGFR to maintain cell survival and promote osimertinib tolerance.^220^ In addition, AXL overexpression is found to enhance DTC survival in EGFR‐mutant lung cancer.^224^ One of the mechanisms for AXL to help DTCs survival is proposed that AXL could bind to the ligand GAS6, while the activation of the GAS6/AXL axis can promote the dormancy of cancer cells.^224^, ^226^

Furthermore, HER3 overexpression is induced by FOXD3 activation, which is caused directly by BRAF or MEK suppression. When its ligand NGB1 is present, HER3 can overcome MEK or BRAF suppression because the AKT survival pathway is activated; however, the treatment with lapatinib, an ERBB2 inhibitor, could counteract the above effect.^227^ HER3 activation contributes to DTCs survival in ALK‐rearranged lung cancer treated with ALK‐TKIs. In comparison with ALK‐TKIs monotherapy, the combined treatment with afatinib, a pan‐HER inhibitor, and ALK‐TKI has the potential to suppress cancer development and eradicate DTCs.^228^

It has been demonstrated that the Notch signaling pathway is one of the most important signaling pathways in drug‐resistant tumor cells.^229^, ^230^, ^231^, ^232^ The Notch signaling pathway is also reported to be involved in the regulation of drug tolerance. For example, Notch signal promotes glioblastoma to enter a DT state after treatment with a kinase inhibitor.^61^ Different genes involved in the Notch pathway, such as downstream targets, notch ligands and receptors, are upregulated in DTCs. Treatment with an inhibitor of γ‐secretase, a protease essential for activating Notch, curbs DTC growth. Rapid activation of Notch3 is found in EGFR‐mutant lung cancer after EGFR TKI therapy, leading to an enhancement in β‐catenin activation and stability, which facilitates DTC survival.^233^

FGFR3 has been shown to prolong the survival of NSCLC patients with mesenchymal EGFR mutations.^116^ Treatment with an EGFR inhibitor can enhance the expression of FGFR3 and other FGF family ligands. In vivo, EGFR inhibitors combined with FGFR inhibitors have been demonstrated to block DTC expansion and restrain their long‐term survival, thus preventing tumor recurrence. In both mouse and human BCC, vismodegib, a smoothened inhibitor, is found to induce the expression of LGR5 in DTCs, which is characterized by the activated Wnt pathway. Ablation of the LGR5 lineage or inhibition of the Wnt pathway in combination with vismodegib therapy is demonstrated to inhibit the survival and expansion of DTCs.^65^ In addition, Eggermont et al.^234^ demonstrated that the EGFR–STYK1–FGF1 axis helps maintain the resistance to EGFR inhibitors in the EGFR‐mutant NSCLC. In gastric cancer, it is reported that the ALDH1A3–mTOR axis facilitates the growth and survival of DTCs after 5‐FU or SN38 treatment.^235^

Numerous intracellular molecules are found to be potential inducers of the DT state. According to a recent study, dysregulation of ufmylation and endoplasmic reticulum stress promotes the survival of DTCs derived from TKI ‐treated PC‐9 cells.^236^ In addition, after EGFR TKI treatment, suppression of ERK1/2 reactivation after inhibition of both EGFR and MEK helps identify the surviving cells, which have progressed into a senescence‐like dormant state and enhanced the YAP/TEAD activity. SLUG, an EMT‐TF, is involved in YAP/TEAD to directly suppress the activity of proapoptotic BMF, thus leading to the inhibition of drug‐mediated apoptosis.^74^ Shah et al.^237^ revealed that activating aurora kinase A (AURKA) in the TKI‐exposed DTCs from EGFR‐mutant NSCLC inhibits drug‐mediated apoptosis by enhancing the phosphorylation and degradation of BIM. The treatment with AURKA combined with EGFR inhibitors could eradicate TKI‐exposed DTCs.^237^

Emran et al.^142^ discovered that after chemotherapy or targeted therapy, tumor cells could survive from the early stress‐mediated DT state by activating the IFN pathway to maintain a slow proliferation rate. NF‐κB signaling is reported to promote lung cancer cell survival and residual diseases in the initial stage of EGFR inhibitor treatment.^238^ It has been reported that the IFN pathway is active in DTCs derived from EGFR‐mutant NSCLC cells. Several IFN response/antiviral defense markers and IRF7 (a critical IFN regulator) are among those significantly induced genes in the DTCs.^45^ Tetsu et al.^239^ found that inactivation of the AKT activity and impairment of the Ets‐1 function caused by EGFR inhibition facilitate the survival of DTCs derived from PC‐9 cells after exposure to EGFR inhibitor treatment. In melanoma, one of the melanoma survival oncogenes, MITF, is found to be the driver for the reversible DT state after exposure to BRAF and MEK inhibitors.^240^, ^241^ In addition, acetylcholine (ACh), a neurotransmitter, is highly expressed in DTCs from EGFR‐mutant NSCLC treated with EGFR TKI. The upregulation of the ACh metabolism promotes drug tolerance partially through activating the WNT signaling via the ACh muscarinic receptor 3 (M3R), thus targeting the ACh/M3R signaling with darifenacin could prevent DTCs formation.^242^

### 3.5 Antiapoptosis

Previous studies have confirmed that apoptosis resistance plays an important role in drug resistance in tumors.^243^, ^244^, ^245^ Of note, suppression of apoptosis has also been found to be associated with drug tolerance and it may be a promising therapeutic approach for DTCs. For example, BCL‐x and BCL‐2 play a key role in preventing cancer cell apoptosis.^246^, ^247^, ^248^ DTCs from EGFR‐mutant NSCLC cells treated with EGFR inhibitors display apoptosis suppression and they become sensitive to the treatment with EGFR inhibitors in combination with navitoclax (ABT293), a BCL‐x, and BCL2 inhibitor.^47^ BIM, a critical proapoptotic protein, is a predictor of the apoptotic response to the treatment in various tumor cells.^215^ Similarly, suppression of Myc or Brd4, a Myc transcriptional coactivator, helps attenuate drug cytotoxicity through hindering the initiation of apoptosis by BIM and BID in the DTCs from BC treated with chemotherapy,^77^ indicating that inhibition of Myc promotes DTC survival by reducing the activity of proapoptotic proteins. In the DTC from prostate cancer cells treated with flavopiridol, a pan‐CDK inhibitor, transcriptomic analysis reveals that mRNA expressions of antiapoptotic factors and apoptosis inhibitors are upregulated, while mRNA expressions of apoptosis‐inducing genes are downregulated.^194^ The DTCs from EGFR‐mutant NSCLC have been shown to be resistant to the treatment by osimertinib and gefitinib via the promotion of the expression of MCL1, an antiapoptotic protein, through mTORC1‐induced mRNA translational regulation at the posttranscriptional level.^137^ In addition, SLUG has been reported to be involved in YAP/TEAD for direct suppression of BMF, a proapoptotic protein, thereby reducing drug‐mediated DTC apoptosis in EGFR‐mutant NSCLC.^74^ Thus, resistance to drug‐induced apoptosis represents one of the adaptation strategies for DTCs to survive from chemotherapy.

### 3.6 TME

It is now evident that the TME plays a pivotal role in tumor growth, progression, metastasis, and response to anticancer treatments.^249^, ^250^, ^251^, ^252^, ^253^, ^254^, ^255^ The TME components consist of an extracellular matrix (ECM) and multiple cell types including cancer‐associated fibroblasts (CAFs), tumor‐associated macrophages (TAMs), endothelial cells, and mesenchymal stem cells (MSCs).^256^ Tumor growth and progression and subsequent development of chemoresistance are mediated by the cross‐talk between tumor cells and their surrounding stromal cells via paracrine loops, chemokine networks, and cell–cell interactions.^257^, ^258^, ^259^, ^260^ CAFs are one of the most important nonmalignant cell populations in the TME, and they play a crucial role in cancer progression, metastasis and chemotherapy resistance.^261^, ^262^ For example, the CAF‐secreted hepatocyte growth factor (HGF) has been reported to mediate EGFR TKI resistance through stimulating the phosphorylation of Met in TNBC.^263^ CAFs could activate the JAK/STAT3 signaling by secreting tumor promoting factors such as IL‐6, and then enhance the expression of drug resistance‐promoting genes, thus promoting chemotherapy resistance.^264^, ^265^ In addition, the cross talk between CAFs and tumor cells via fibronectin, fibroblast‐derived factor (FGF) and β1 integrin could activate PI3K/AKT and MAPK/ERK 1/2 pathways that lead to drug resistance.^266^, ^267^ In addition to secreting growth factors, CAFs can remodel the local stromal microenvironment to promote cancer cell survival during chemotherapy treatment.^268^ TAMs, another important nonmalignant cell population in the TEM, also mediate tumor growth, development, and metastasis, as well as therapeutic responses.^269^, ^270^, ^271^ Through secreting survival factors and/or activating antiapoptotic signaling pathways, TAMs could promote drug resistance in tumor cells.^272^, ^273^ For example, TAM‐derived IL‐10 promotes paclitaxel resistance in human breast tumor cells by activation of the STAT3 signaling and upregulation of bcl‐2 gene.^274^ In addition, endothelial cells,^275^, ^276^ MSCs,^277^, ^278^ and the ECM^279^, ^280^, ^281^ have been found to contribute to drug resistance of cancer cells. CSCs play an influential role in tumor progression, recurrence, and chemoresistance due to their typical stemness characteristics. The TME components of CAFs, TAMs, MSCs, and noncellular factors have been reported to promote tumor cell stemness via bidirectional cross talks between tumor cells and secreted factors.^282^ For example, CAFs could promote tumor stemness through the FGF4–FGF2 axis and high‐mobility group box 1.^283^, ^284^ In addition, immune cells in the TME may regulate stemness and self‐renewal of tumor cells via cytokine networks.^285^, ^286^

It has been confirmed that the TME can promote tumor drug resistance, but the effect of the TME on drug tolerance remains to be unveiled. It has been reported that TKI treatment indirectly induces phenotypic changes in CAFs, which could promote DTC survival through STAT3 activation in lung cancers with EGFR mutations.^287^ Hirata et al.^251^ reveal the relationship between the TME and drug tolerance in BRAF‐mutant melanoma treated with a BRAF inhibitor (BRAFi) through intravital imaging of cells via an ERK/MAPK biosensor: the inhibition of BRAF leads to paradoxical activation of CAFs and matrix remodeling. In addition, an increase in the ECM stiffness activates the integrin, FAK, and Src signaling, ultimately melanoma cells become DT by reactivation of the MAPK signaling. Inhibition of this signaling pathway resensitizes melanoma to BRAF inhibitors, suggesting that therapeutic strategies that target stromal–tumor interactions may have the potential to overcome drug resistance.^251^

As discussed above, many mechanisms for the development of the DT state have been proposed, and DTC treatment has yet to be established.

## 4 THERAPEUTIC OPPORTUNITY

On the basis of understanding the characteristics of DTC and its related mechanism, researchers have proposed potential therapeutic strategies for targeting DTCs. The first one is to maintain tumor cells in a DT state, and the second is to target specific regulators. After prolonged treatment, tumor cells in a tolerant state may finally develop into stable genetically‐resistant clones through a variety of mechanisms, so the first approach may not be feasible in the clinical practice. In contrast, specifically targeting DTC regulators could be a potentially efficacious and viable treatment option. This approach can be realized through targeting factors for maintaining the DT state, or prevention of tumor cells from entering the DT state. Nonetheless, both of them are involved targeted therapy with anti‐DTC factors combined with standard antitumor agents (Table 1).

**TABLE 1:** Studies on drug‐tolerant cells to elucidate mechanisms of tolerance via various cancer types and models.

Cancer type	Model	Initial treatment	Mechanism of DTC	Target of therapy	Drugs targeting DTCs	References
NSCLC	PDC(PC9)	EGFR TKI‐Gefitinib	Epigenetic modifications and transcriptional regulation	KDM5A	HDAC inhibitor (TSA)	^44^
Glioblastoma	PDC (GSC8, CW1691)	PDGFR inhibitor‐Dasatinib	Epigenetic modifications and transcriptional regulation	KDM6	KDM6 inhibitor (GSKJ4)	^61^
TNBC	PDX, PDC (MDA‐MB‐468)	Capecitable	Epigenetic modifications and transcriptional regulation	KDM6	KDM6 inhibitor (GSKJ4)	^66^
NSCLC, CRC, melanoma,breast cancer	PDC (PC9, colo205, M14, SKBR3, EVSA‐T)	Target therapy (Erlotnib, AZ628, lapatinib, PI3 kinase inhibitor)	Epigenetic modifications and transcriptional regulation	KDM5	KDM5 inhibitor (CPI‐455)	^141^
NSCLC	PDC(PC9)	EGFR TKI‐Gefitinib	Epigenetic modifications and transcriptional regulation	KDM5	Ryuvidine	^142^
NSCLC	PDC(PC9)	EGFR TKI‐Erlotinib	Epigenetic modifications and transcriptional regulation	H3K9 methylation over LINE‐1 elements	HDAC inhibitor (MS275 or TSA)	^151^
Breast cancer	PDC (HCC1143, SUM149PT)	MEK and PI3K/mTOR inhibitor (BEZ235 and Trametinib)	Epigenetic modifications and transcriptional regulation	BET	BET inhibitor (JQ1)	^161^
TNBC	PDX	Doxorubicin, cyclophosphamide	Metabolic changes	Oxidative phosphorylation	Oxidative phosphorylation inhibitor (Oligomycin)	^53^
NSCLC, GC	PDC (PC9, MKN45)	EGFR TKI‐Erlotinib, crizotinib	Metabolic changes	ALDH	ALDH inhibitor (Disulfiram)	^59^
NSCLC, ovarian cancer, breast cancer, melanoma	PDC (PC9, A375, BT474, Kuramochi)	TKI, chemotherapy	Metabolic changes	GPX4	GPX4 inhibitor (RSL‐3, ML20)	^60^
CRC	PDX	5‐Fluorouracil, leucovorin, irinotecan	Metabolic changes	Autophagy pathway (ULK1)	Autophagy/ULK1 inhibitor (SBI‐ 0206965)	^67^
Melanoma	PDC(WM3734)	Vemurafenib, chemotherapy	Metabolic changes	Oxidative phosphorylation	Oxidative phosphorylation inhibitor (Oligomycin)	^70^
Breast cancer, prostate cancer	PDC (Du145, MCF‐7)	Taxol, adriamycin	Metabolic changes	NPC1L1	NPC1L1 inhibitor (Ezetimibe)	^198^
Pancreatic cancer	PDX	K‐ras inhibitor	Metabolic changes	Oxidative phosphorylation	Oxidative phosphorylation inhibitor (Oligomycin)	^209^
LUAD	PDC and PDO	MAPK inhibitor	Metabolic changes	Mitophagy	Chloroquine	^212^
NSCLC	PDC(PC9)	EGFR TKI‐Gefitinib	Signaling pathway	IGF‐1R signaling	IGF‐1R inhibitor (AEW541)	^44^
Melanoma	PDX	Raf /MEK inhibitor‐Dabrafenib/trametinib	Signaling pathway	RXR signaling	RXR inhibitor (HX531)	^50^
BBC	PDX	Hedgehog inhibitor‐Vismodegib	Signaling pathway	Wnt signaling	Wnt signaling inhibitor (LGK‐974)	^65^
NSCLC	PDC (PC9, HCC4006)	EGFR TKI‐Osimertinib trametinib	Signaling pathway	YAP‐TEAD signaling	TEAD inhibitor (MYF‐01−37)	^74^
Breast and prostate cancer	PDO, PDX	Docetaxel	Signaling pathway	Myc	CDK9 inhibitor	^77^
NSCLC	PDC (PC9, H1975, HCC827, HCC4006)	EGFR TKI‐Gefitinib, osimertinib	Signaling pathway	FGFR3 signaling	pan‐FGFR inhibitor (infigratinib)	^116^
NSCLC	PDC (H2228, A925L)	ALK‐TKIs	Signaling pathway	HER3	pan‐HER inhibitor (afatinib)	^211^
NSCLC	PDC (PC9, HCC4011)	EGFR TKI‐Osimertinib	Signaling pathway	AXL signaling	AXL inhibitor (NPS1034)	^220^
NSCLC	PDC (HCC827, HCC4006)	EGFR TKI‐Erlotinib	Signaling pathway	WNT/β‐catenin signaling	β‐catenin inhibitor (ICG‐001, XAV939)	^233^
GC	PDC	5‐Fluorouracil	Signaling pathway	mTOR	mTOR inhibitor (Temsirolimus)	^235^
NSCLC	PDC (PC9, H1975)	EGFRTKI‐Osimertinib, Rosiletinib	Signaling pathway	AURKA	Aurora kinase inhibitor (MLN8237)	^237^
NSCLC	PDX	EGFR TKI‐Erlotinib	Signaling pathway	NF‐κB signaling	PBS‐1086	^238^
Melanoma	PDC (A375, Malme‐3 M and WM983B)	BRAF(PLX4032) and MEK (cobimetinib) inhibitors	mRNA translation remodeling	eIF4A	eIF4A inhibitor (silvestrol)	^190^
Melanoma	MDC (5555,4434)	Raf inhibitor‐PLX4720	TME	Integrin β1/FAK signaling	FAK inhibitor (PF562271)	^251^

DTC, drug‐tolerant cell; PDC, patient‐derived cell lines; PDX, patient‐derived xenografts; PDO, patient‐derived organoids; MDC, mouse‐derived cell lines; KDM5/6, lysine‐specific demethylase 5/6; HDAC, histone deacetylase; LINE‐1, long interspersed repeat element 1; GPX4, glutathione peroxidase 4; ALDH, aldehyde dehydrogenase; ALK, anaplastic lymphoma kinase; AURKA, aurora kinase A; AXL, Axl receptor tyrosine kinase; NSCLC, non‐small‐cell lung carcinoma; BCC, basal cell carcinoma; GC, gastric cancer; CRC, colorectal cancer; TNBC, triple‐negative breast cancer; EGFR, epidermal growth factor receptor; FAK, focal adhesion kinase; FGFR, fibroblast growth factor receptor; HER3, human epidermal growth factor receptor 3; MET, tyrosine‐protein kinase Met or hepatocyte growth factor receptor; PI3K, phosphoinositide 3 kinases; IGF‐1R, insulin‐like growth factor 1 receptor; TKI, tyrosine kinase inhibitor; TME, tumor microenvironment.

### 4.1 Master regulators of biological processes

Various strategies by targeting epigenetic modifiers, and transcription and translation factors can be used to eliminate DTCs. KDM5A is found to be upregulated in the DTCs from EGFR‐mutant NSCLC cells; therefore, DTCs may be eradicated using EGFR TKI in combination with a HDAC inhibitor or a KDM5A inhibitor.^44^, ^140^ In glioblastoma, The DTCs obtained after persistent MEK inhibition show preferential GSKJ4 sensitivity, and DTCs death is achieved by using a small‐molecular GSKJ4‐KDM6A/B inhibitor.^61^ In addition, a KDM6 inhibitor inhibits the formation of DTCs in TNBC following the administration of a chemotherapeutic agent.^66^ EZH2 inhibition, which results in the loss of H3K27me3, also helps reduce the number of DTCs derived from NSCLC.^151^ DTCs derived from lung cancer show a global decrease in the H3K acetylation level.^45^ Consistently, treatment with HDAC inhibitors, such as MS275 and trichostatin A, in combination with targeted therapy leads to selective ablation of DTCs derived from lung cancer either in vivo or in vitro.^44^, ^151^ Similarly, DTCs exhibit a suppressed chromatin state when the H3K9/H3K27 methylation level of the repetitive element LINE‐1 is enhanced, and downregulation of LINE‐1 by HDAC inhibitors could promote the chromatin accessibility, finally resulting in DTC ablation.^45^ In addition, m6A‐associated mRNA translation remodeling is reported to sustain the survival of melanoma persister cells upon BRAFi/MEKi treatment, and inhibition of translation remodeling using eIF4A inhibitors can help eradicate melanoma persister cells.^190^

### 4.2 Metabolic enzymes

Because DTCs rely on an antioxidant mechanism and mitochondrial respiration to mitigate the resulting oxidative stress, DTCs may be susceptible to inhibitors for GPX4 and mitochondrial OXPHOS.^60^, ^70^, ^209^ Several DTC experimental models for BC, lung cancer, CRC, and gastric cancer after targeted treatment reveal that the expression of aldehyde dehydrogenase is essential for protecting DTCs from ROS‐induced toxicity.^59^ In a phase‐2b clinical trial, ALDH serves as an antioxidative stress mechanism, and the treatment with an ALDH inhibitor combined with disulfiram and cisplatin‐based chemotherapy achieves enhanced survival of advanced NSCLC patients.^288^ In addition, DTCs overcome oxidative stress induced by chemotherapy partially through enhancing the NPC1L1‐modulated vitamin E absorption. Treatment with ezetimibe, a NPC1L1 inhibitor, and chemotherapeutic agents could enhance the therapeutic effect of the combination therapy through inducing mehuosis.^198^ Diapause‐like DTCs in the CRC models are seen to rely on an upregulating autophagy procedure. The treatment with CPT‐11 combined with chloroquine, a global autophagy inhibitor, significantly induces apoptosis compared with monotherapy with CPT‐11.^67^ Furthermore, PINK1‐mediated mitophagy promotes DTCs survival. Mitophagy inhibition through either employment of chloroquine or depletion of PINK1 could help eradicate drug tolerance.^212^

### 4.3 Signaling transduction molecules

Multiple signaling pathways have been reported to sustain DTC survival, so targeting a signaling pathway is an effective treatment method. In EGFR‐mutant lung cancer, inhibition of IGF‐1R, AXL, FGFR3, YAP‐TEAD, AURKA, Wnt/b‐catenin, and NF‐κB transcription in the DTCs combined EGFR TKI treatment will help eliminate DTCs.^44^, ^74^, ^116^, ^220^, ^233^, ^237^, ^238^

In ALK‐rearranged lung cancer, it has been reported that HER3 activation contributes to maintaining cell survival while inducing ALK‐TKI‐DTC occurrence, whereas the treatment with afatinib, a pan‐HER inhibitor, combined with ALK‐TKIs could suppress tumor growth and eventually eliminate DTCs.^228^ In breast and prostate cancer, Myc‐suppressed diapause‐like cells are found to maintain a DT state and display persistence to therapeutic treatments; and CDK9 inhibition reverses this state and helps restore their sensitivity to chemotherapeutics.^77^ In gastric cancer, exposure to Temsirolimus, an mTOR inhibitor, eradicates 5FU‐tolerant cancer cells through inhibition of the ALDH1A—mTOR axis.^235^ Sanchez‐Danes et al.^65^ discovered that slow‐cycling LGR5+ tumor cells promote BCC recurrence after vismodegib (an Hh inhibitor) treatment. Notably, activation of the Wnt pathway is found in such persistent, slow‐cycling LGR5‐expressing tumor cells, and inhibition of both Wnt and Hh is successful in suppressing tumor development.^65^ In addition, there are different DT transcriptional states, including a neural crest stem cell‐like (NCSC) transcriptional procedure which is heavily dependent on RXR, a nuclear receptor. Through targeting the RXR signaling via HX531, a selective antagonist, only a small number of NCSC‐like cells are seen after MAPK inhibitor exposure, thereby sensitizing melanoma to conventional therapeutic modalities.^50^

Because DTCs can resist apoptosis and highly express antiapoptotic proteins including BCL‐2, BCL‐XL, and BCL‐W, ABT263 (navitoclax), which can specifically inhibit these antiapoptotic proteins, has been used to kill drug‐mediated senescent tumor cells.^289^ Targeting the TME may also be an effective strategy. Inhibiting the production of HGFs in CAFs can induce the resensitization of cancer cells to gefitinib in EGFR‐mutant NSCLC persisters both in vivo or in vitro.^290^ CAFs can modulate drug sensitivity and the EMT process in a paracrine manner through the activation of IGF‐1/IGF‐1R and HGF/c‐met pathways in NSCLC. Simultaneous inhibition of the above pathways plus the treatment with EGFR TKI contributes to preventing CAF‐mediated drug tolerance and EMT.^291^ Moreover, BRAF‐mutant melanoma cells display initial response to BRAFi treatment, but they gain tolerance to BRAFi quickly after contacting with stromal cells. From a mechanism perspective, melanoma‐associated fibroblasts are responsive to BRAFi, leading to a decrease in fibronectin generation, which promotes FAK signaling in the melanoma cells. Inhibition of both BRAF and FAK suppresses MAPK reactivation, thus controlling tumor development.^251^ Therefore, targeting the TME may also be an effective strategy for eradicating DTCs.

## 5 CHALLENGES AND PERSPECTIVES

### 5.1 Origins of DTCs

**FIGURE 5:**
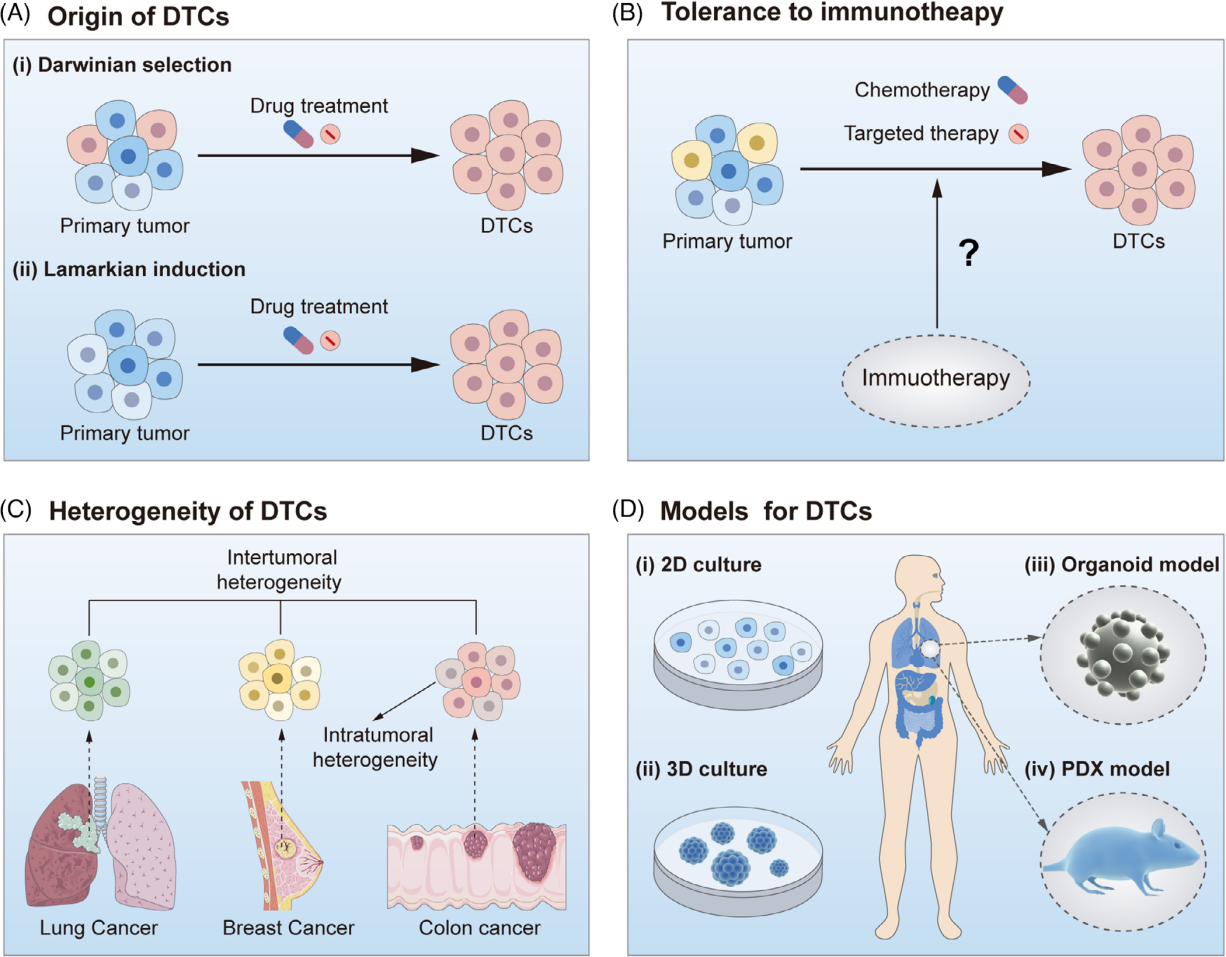
Challenges of the DTC‐targeting approach to overcome acquired drug resistance. (A) Origin of DTC (Darwinian selection or Lamarkian induction). (B) Tolerance of tumor cell to chemotherapy or targeted therapy has been confirmed, while tolerance to immunotherapy remains unclear. (C) Intra‐ and intertumor DTC heterogeneity may exist. (D) The present models used for DTC include 2D culture, 3D culture, organoids and PDX models. DTC, drug‐tolerant cell; PDX, patient‐derived xenografts.

DTCs may be the source of acquired drug resistance; therefore, unveiling of the mechanism of DTCs generation is critical for developing specific anti‐DTC treatments and inhibiting or delaying the production of DTCs. There is a hot debate over the origin of DTCs. Two dominant hypotheses have been proposed (Figure 5A): First, tumor cells with DTC characteristics may present in untreated tumors, contributing to their selective survival from drug treatment. For example, Kurppa et al.^74^ applied DNA barcoding of PC9 cells exposed to MEK inhibitors alone or in combination. It is found that the majority of the common barcodes in the cells exposed to monotherapy with an EGFR inhibitor match well with those in preexisting persister clones. Shaffer et al.^292^ reported that human BRAF‐mutant melanoma cells display single‐cell transcriptional heterogeneity, which results in transient upregulation of several resistance genes in a small portion of cells, such as AXL, WNT5, EGFR, JUN, and PDGFRB, leading to development of drug resistance and survival of cancer cells from vemurafenib therapy. In addition, pretreatment with RSL3 (a GPX4 inhibitor) decreases the number of persister cells in HER2‐amplified BC BT474 parental cells, which have survived from lapatinib (a HER2 inhibitor) treatment, indicating the presence of GPX4‐sensitive cells in the persister cells.^60^ Untreated oral squamous cell carcinoma (OSCC) cells contain CSC‐like cells, which exhibit KDM5B upregulation, suggesting that DTCs might have presented in the original OSCC cell bank.^293^ The above results are in alignment with those of previous studies to support the conclusion: the presence of persister cells prior to drug exposure.^59^, ^60^ Taken together, a small number of cancer cells in a DT state are present before drug treatment and enriched after drug exposure, which follow the nongenetic Darwinian selection principle.

In addition, drug exposure induces the change in the phenotype from cancer cells to DTCs. Individual cancer cells might become persistent upon exposure to a treatment, via a Lamarkian type of adaptation.^294^ These cancer cells do not have the capacity of drug persistence before the treatment, but drug persistence in these cells can be induced after the treatment. In the aforementioned study conducted by Kurppa et al.^74^ on DNA barcoding, the majority of unique barcodes are seen after treatment with EGFR combined with a MEK inhibitor, which suggests that cells could enter a dormancy state after simultaneous inhibition of both EGFR and MEK. Risom et al.^118^ reported that persister cells in a DT state in BC are enriched from different cell state transition processes that are involved in dynamic open chromatin structural remodeling after mediation of PI3K/mTOR and a MEK inhibitor. The finding cannot be simply explained by Darwinian selection of preexisting subpopulations. Instead, this study supports an “equipotent” model: cancer cells had the same ability to exit and enter the DT state for their survival, which is dramatically different from the clonal selection hypothesis that a small subpopulation of parent tumor cells can become DTCs.^44^

The above two hypotheses about the DTC generation process may complicate the development of anti‐DTC treatment methods. For the clonal selection hypothesis, interventions may be exerted in the initial therapy. Thorough analysis of the samples prior to drug treatment at both transcriptional and translation levels can provide great insights into cell subpopulations, especially those that evolve into DTCs. For the second hypothesis that phenotypic transition of tumor cells into DTCs is induced after drug exposure, the corresponding treatment should be focused on residual diseases, and analysis of posttreatment samples could reveal detailed information on genetic changes of cancer cells.

### 5.2 DTCs after immunotherapy

Immunotherapy has gained popularity to treat tumors because of its excellent antitumor effect, while immunotherapy resistance compromises its therapeutic effect.^295^, ^296^, ^297^ As discussed above, tumor cell tolerance to chemotherapy or targeted therapy has been extensively studied. These studies, however, do not consider that DTCs must evade or be tolerant to immune surveillance in order to survive in the body. The mechanisms of immune tolerance or immune evasion developed by DTCs remains to be unveiled. In addition, it remains unclear whether tumor cells also enter a similar DT state after immunotherapy, such as treatment with immune checkpoint inhibitors (Figure 5B). Given the popularity of immunotherapy against tumors, efforts into demystifying tumor immunotherapy tolerance should be made for better immunotherapeutic effect. It has been reported that tumor cells could exploit multiple mechanisms of escaping immune surveillance, such as upregulating the expression of the major histocompatibility complex (MHC) or downregulating the expression of negative immune checkpoint factors.^298^ For example, after the ACT immunotherapy, stem cell‐like squamous cell carcinoma cells upregulate CTLA4 through the expression of CD80, thereby directly inhibiting cytotoxic T cell activity.^299^ These previous studies have laid a solid foundation for mitigating immunotherapy tolerance in cancer cells.^78^

### 5.3 Heterogeneity of DTCs

It has been demonstrated that critical drivers, environments, and pathways have been identified as DTC regulatory factors. Because the DTC maintenance mechanism differs between tumor types, it is unclear whether DTCs have tumor‐specific or global characteristics. If DTCs are tumor specific, DTCs in specific tumors should be considered to develop anti‐DTCs therapeutic drugs. Furthermore, there may be heterogeneity in the DTC population (Figure 5C). The heterogeneity of DTCs could complicate the method to eliminate them. Most of the current available drugs for eliminating DTCs may not be able to completely eliminate them due to their heterogeneity, and eventually stable resistance could be developed. Single cell sequencing can be used to detect the heterogeneity of DTCs, and therapeutic drugs targeting different characteristics of DTCs can be developed. Moreover, the relationships between various DTC maintenance mechanisms and signaling pathways have not been completely unveiled. For example, in EGFR‐mutant lung cancer, IGF‐1R signal transduction required for the DT state is regulated via KDM5A demethylation. Meanwhile, treatment with an IGF‐1R inhibitor significantly reduces KDM5A expression, indicating that the IGF‐1R pathway is related to the KDM5A activity.^44^

### 5.4 Models for DTCs

The DTCs in the majority of previous studies are produced in vitro from commercially available cancer cell lines; however, such 2D cell culture models may not capture the effect of the TME on the generation of DTCs. Furthermore, little is known about the tumor cell subpopulation in vivo that evolves into DTCs, as well as the critical mechanisms that promote the DTC state in vivo. First, the nature and role of DTCs in clinical MDR remain to be unveiled due to the challenge in acquiring tumor tissues from cancer patients. The approach to obtaining sufficient biopsies from the minimal residual disease of cancer patients should be explored. In addition, construction of in vivo models, such as the PDX model, is expensive and time consuming, and the isolation of proteins and nucleic acids from these persister tumors is challenging because the cancer cell number decreases while the mouse stromal cell number increases. Therefore, robust preclinical models that can be used to study the effect of the TME on DTCs generation should be developed. Great attentions have been paid to organoid models due to their capability of reproducing histological and genetic features of parental tumor cells, and they can be manipulated to reveal the mechanisms of DTCs generation and eradication. Since no stromal cells are incorporated in the tumor organoid model, recent studies have explored to build a TME in the organoid model through coculture of cancer cells with immune cells and fibroblasts, hopefully this model can be used to reveal the effect of the immune system and the microenvironment on DTCs generation.^300^ These organoids model may also be employed to reveal DTC characteristics and regulatory mechanisms in cancer patients (Figure 5D).

## 6 CONCLUSION

Drug resistance remains a main challenge in antitumor therapy. During early drug treatment, cancer cells would enter a DT state to escape from drug toxicity, and these cells become a reservoir for drug resistance and tumor recurrence and metastasis, as a result, these cells are the promising targets to overcome drug resistance. Previous studies have discovered multiple mechanisms and therapeutic approaches for treating DTCs, but none of these approaches have been clinically translated. Successful strategies of targeting DTCs requires a deeper understanding of the mechanisms that drive the DT state in tumors in a more specific clinical context. One of the priorities of future studies is to develop a feasible approach to obtaining adequate biopsies from cancer patients with the minimal residual disease. In addition, development of methods for tissue‐based profiling, such as highly multiplexed immune fluorescence, mass spectrometry, and spatial transcriptomics, and single‐cell sequencing can help us reveal the origin, characteristics, heterogeneity and regulatory mechanisms of DTCs, and provide great insights into the development of therapeutic methods to target DTCs and overcome tumor resistance. In addition, tumor cells can maintain drug resistance through a variety of mechanisms, while most of current therapies only target one of these mechanisms. Therefore, new therapeutic strategies such as combination therapy by target multiple key signaling pathways could be employed to overcome drug resistance.

## AUTHOR CONTRIBUTIONS

Xiaohai Song, Yang Lan, and Xiuli Zheng worked together to perform the literature search, study design, and writing; Qianyu Zhu, Xuliang Liao, Kai Liu, Weihan Zhang, QiangBo Peng, and Yunfeng Zhu helped to prepare the figures; Linyong Zhao, Xiaolong Chen and Yang Shu helped to prepare the table and discussed the concepts of the manuscript; Kun Yang and Jiankun Hu performed the supervision and revision. All authors read and agreed to the published version of the manuscript.

## CONFLICT OF INTEREST STATEMENT

All authors declare that they have no conflicts of interest.

## ETHICS STATEMENT

Ethics approval was not needed for this study.

## Data Availability

Not applicable.
